# Imaging Techniques for the Study of Protein Condensates and Filaments and Their Applications

**DOI:** 10.3390/ijms27073063

**Published:** 2026-03-27

**Authors:** Xiaotang Shen, Yueyang Liu, Yan-Wen Tan

**Affiliations:** State Key Laboratory of Surface Physics, Shanghai Key Laboratory of Metasurfaces for Light Manipulation, Department of Physics, Fudan University, Shanghai 200433, China; 23110190060@m.fudan.edu.cn (X.S.); 21210190050@m.fudan.edu.cn (Y.L.)

**Keywords:** phase separation, protein condensates, protein filaments, foci

## Abstract

Protein condensates and filaments are both intracellular structures characterized by their ability to facilitate specific biological functions. Their formation is primarily driven by phase separation, which can be elucidated by fluorescence microscopy or electron microscopy. Here we summarize the main studies on protein condensates and filaments organized according to the techniques used, including fluorescence methods like localization screening, fluorescence co-localization spectroscopy, methods based on photobleaching, super-resolution imaging, and electron methods including negative-stain electron microscopy and cryo-EM. We also discuss correlative light/electron microscopy (CLEM), which integrates fluorescence microscopy and electron microscopy to provide complementary insights. Collectively, these methods offer temporal and spatial insights into investigating the phase separation of protein condensates and filaments, and promote the discovery of unexplored structures and their yet-to-be-characterized biological roles.

## 1. Introduction

Since the early 21st century, many intracellular structures have been identified, such as P bodies [1,2], stress granules [3], U bodies [4], and purinosomes [5]. In 2009, a new intracellular structure was found: Narayanaswamy et al. found that 33 proteins were capable of forming punctate structures upon nutrient starvation [6]. This was followed in 2010 by an independent report from three groups that Ctps, a metabolic enzyme for synthesis of the nucleotide CTP, could form filaments [7,8,9], a structure which was named cytoophidium. These discoveries ignited a wave of scientific exploration into their assembly mechanisms, biological functions, cellular significance, and dynamic regulation.

Previous studies have found that many proteins could form filamentous structures. These assemblies were imaged in vitro through techniques such as electron microscopy (EM) [10,11,12] and fluorescence microscopy (FM) [13,14]. Crucially, although these observations captured what we now recognize as prototypical protein condensates or filaments, they were not investigated within the corresponding conceptual framework. The systematic study of protein condensates or filaments now provides a unified lens for reinterpretation of these historical findings.

The punctate structures observed in fluorescence microscopy are termed protein condensates, which are defined as membraneless, non-stoichiometric cellular assemblies that are composed of multiple types of biomolecules, occur through phase transitions, and can be investigated by using concepts from soft matter physics [15,16]. These structures have two common features: they contain biological molecules, and they have the ability to concentrate molecules [17]. The filamentous structures observed in fluorescence microscopy are termed protein filaments. Morphologically, protein filaments can adopt linear, curved, or helically arranged conformations and may appear as single-stranded or multi-stranded polymers. In many cases, these filaments are distinct from the canonical cytoskeleton and frequently consist of, or contain, metabolic enzymes [18]. For protein condensates, the high-volume spherical bodies are more suitable for storage [19], which enable them to contain translational factors [20]. Protein filaments, while more responsive to external stimuli [19], contain a limited number of enzymes [20].

The formation of these condensates or filaments is generally considered to be driven by phase separation [21], but whether these phases are solid or liquid depends on the proteins themselves. Saad et al. found that Cdc19 condensates, one of the pyruvate kinase paralogs, were solid-like using FRAP [22]. Prouteau and Loewith pointed out that since some condensates can be separated by ultra-centrifugation, these condensates are solid [21]. Banani et al. suggested that these condensates are highly mobile molecules that can exchange with surrounding cytoplasm [17].

Phase separation of protein condensates or filaments is a developing area. Reviews of phase separation primarily focus on liquid–liquid phase separation forming foci, yet exclude filamentous structures such as cytoophidium. Only a few reviews introduce the phase separation of protein condensates or filaments [18,20,21,23,24,25,26,27]. In our review, we focus on the technical aspects of protein condensate or filament studies. By summarizing methods to investigate these protein structures, we can gain new insights and ideas for the study of new systems. The fluorescence methods discussed below include localization screening, fluorescence co-localization microscopy, methods based on photobleaching, and super-resolution imaging. These fluorescence methods offer unparalleled capabilities for live-cell, time-resolved imaging, and high-throughput screening, but are limited by spatial resolution. Electron microscopy approaches comprise negative-stain electron microscopy and cryo-EM. Electron microscopy provides near-atomic detail on ultrastructure but is limited to in vitro experiments, and fails to offer time-resolved information. In addition, we introduce works conducted via correlative light/electron microscopy (CLEM), which combine fluorescence microscopy and electron microscopy. Our aim is to provide a methodological framework for the investigation of protein condensates and filaments, from initial discovery to structural and functional elucidation.

## 2. Fluorescence Method for Observing Protein Condensate or Filament

Although the earliest discoveries of protein filaments were performed via electron microscopy, since the advent of fluorescent proteins, fluorescence microscopy has become the main powerhouse for the identification of new protein condensates or filaments. Among all the common imaging methods, fluorescence microscopy is relatively cost-effective and there is a wealth of versatile probes to choose from. Using protein fluorescence or immunofluorescence, fluorescence microscopy can observe condensate- or filament-forming proteins both in vivo and in vitro. Fluorescence microscopy can be conducted in a time-lapse manner with the time resolution determined by the imaging instrument, i.e., CCD cameras or photon detectors. For in vivo experiments, researchers can observe these proteins under different cell growth phases or nutrient conditions.

Fluorescence microscopy can be applied to observe the dynamic processes of the condensates or filaments. Petrovska et al. took a video of the Gln1 filament formation process in starved yeast [28]. The Gln1 filament started forming after starvation for 50 min. Different cells react to starvation differently, but a drop in pH seems to be the common factor for filament formation. After 18 min of the addition of nutrients, the filaments dissolved. Gou et al. recorded the fusion of cytoplasmic-cytoophidia and nuclear-cytoophidia, separately [29]. For C-cytoophidia, initially, the GFP signal diffused. Then within 5 min, linear cytoophidia formed. The fusion could either be end-to-end or side-by-side. For N-cytoophidia, within 15 min, dot-like cytoophidia formed. Both N- and C-cytoophidia could start forming by the fusion of smaller condensates. Prouteau et al. recorded the process of the protein kinase target of rapamycin complex 1 (TORC1) foci formation and diffusion during glucose deprivation and glucose recovery [30]. After two and a half minutes of glucose deprivation, TORC1 foci became apparent. After 10 min of glucose addition, the majority of foci disappeared. Saad et al. observed the process of Cdc19 foci formation and diffusion during nutrition deprivation and nutrition recovery [22]. Within 4 h of starvation, about 80% of the cells exhibited foci. These foci disappeared rapidly after glucose addition. Stoddard et al. observed Glk1 foci formation and diffusion during glucose addition and deprivation [31]. Glk1 diffused under starvation. After glucose addition, Glk1 filaments formed rapidly.

The above are examples of fluorescent observation extended through a certain time period. In addition to time-lapsed dynamic observations, easier sample preparation and high-throughput imaging enable localization screening for potential condensate- or filament-forming proteins. Co-localization techniques enable researchers to identify the interaction between these enzymes. Methods based on fluorescence photobleaching can measure the mobility of these proteins. These methods will be introduced in Section 2.1, Section 2.2, Section 2.3 and Section 2.4.

### 2.1. Localization Screening for Condensate- or Filament-Forming Proteins

In 2003, Huh et al. created the budding yeast *S. cerevisiae* GFP strain collection [32]. The budding yeast GFP strain collection is aimed at providing proteins’ localization and defining their functions in the context of yeast cellular compartments. This GFP strain collection contains 4159 strains covering 75% of the proteome. The information is available at https://www.yeastgenome.org/ (accessed on 14 March 2026).

Based on the budding yeast GFP strain collection, localization screening for condensate- or filament-forming proteins began in 2009. Narayanaswamy et al. first found that some proteins could form reversible condensates inside quiescent cells [6]. They examined changes in the localization of the GFP-tagged yeast strain collection under nutrition starvation, and identified 33 proteins capable of forming condensates. In 2010, Noree et al. performed a more extensive screening of the yeast GFP strain collection [7]. They screened the collection of 1632 strains of the budding yeast with GFP fused to the C terminus of a single protein. Nine proteins were identified that could form four distinct filaments, and 29 proteins could localize to discrete cytoplasmic foci but could not form filaments. Furthermore, they focused more on the potential biochemical functions of these protein condensates or filaments, especially the filament formed by protein CTPS, which was discovered independently by three different groups on different organisms [7,8,9]. In 2014, O’Connell et al. found that the condensates were cytoplasmic, insoluble protein assemblies [33]. They tested the condensate-forming condition of these proteins, such as heat shock and chemical stress. A total of 117 proteins became insoluble under heat shock, and 143 proteins became insoluble after arsenic treatment, 59 of which were cytoplasmic proteins. In 2016, Shen et al. screened all 4159 strains of the budding yeast to find additional filament-forming proteins [34]. They identified 23 proteins, including nine proteins and four septin proteins that were identified in 2010. They could form filaments in vivo in diauxic and stationary phases. They also analyzed the relationship between these proteins. In 2019, Noree et al. tried to identify more condensate- or filament-forming proteins [35]. With more sophisticated high-throughput methods, they resolved fundamental questions left unanswered by their former work; for example, the rules for these proteins to form condensates or filaments. They measured the frequency of all 440 proteins in the yeast GFP strain collection under log phase, postdiauxic shift, and stationary phase. Their screening identified 60 proteins capable of forming condensates or filaments. The screening for condensate- or filament-forming proteins laid a solid basis for further research on these proteins.

Subcellular localization screenings of other organisms were also carried out. In 2009, Werner et al. performed a high-throughput imaging of *Caulobacter* proteins [36]. Ctps were found to form filaments in *Caulobacter crescentus* in 2010 [8]. In 2014, Lowe et al. established *Drosophila* protein subcellular localization by using large-scale protein trap screens using pigP protein trap library [37]. The subcellular localization of *Caenorhabditis elegans* proteins was performed in body wall muscles [38] and in neurons [39].

As cell growth phase, components of culture medium, and preparation of cells for microscopy examination influence the condensate or filament formation [40], these screening may not include all condensate- or filament-forming proteins. Furthermore, some proteins may not form specific structures in budding yeast, but can form condensates or filaments in other organisms. For example, for IMPDH, a GTP synthesis-related enzyme, filaments are only found in mammalian cells [41].

### 2.2. Fluorescence Co-Localization Spectroscopy

Fluorescence co-localization spectroscopy is an approach for the visualization of protein–protein interactions [42]. Proteins of interest (POI) are tagged with fluorescence proteins or other fluorescent probes of different colors. If protein–protein interaction exists, different signals from the same position can be detected. The determination of “co-localization” is, of course, limited by the spatial resolution of the fluorescent imaging method applied. In conventional fluorescent microscopy, this is restricted by diffraction limit (~200 nm). However, when the labeled protein of interest (POI) is densely distributed within the field of view and multiple molecules overlap within a diffraction-limited spot, methods such as Förster resonance energy transfer (FRET) [43,44] or bimolecular fluorescence complementation (BiFC) [42,45] should be employed for more reliable detection of protein–protein interactions. FRET exploits the phenomenon whereby two proximal fluorophores exchange energy: a donor fluorophore (with higher emission energy and shorter emission wavelength) transfers its excitation energy non-radiatively to an acceptor fluorophore (with lower energy and longer emission wavelength) in a distance-dependent manner. Consequently, excitation of the donor yields a detectable fluorescence signal from the acceptor, indicating close spatial proximity of the labeled proteins—a hallmark of interaction. Conventional FRET dye pairs typically have a characteristic distance known as Förster radius (R_0_), which is typically around 5 nm. This allows FRET to provide substantially higher distance resolution (±0.5 R_0_) than co-localization within the ~200 nm diffraction limit. BiFC relies on reconstituting fluorescence: non-fluorescent split fragments of a fluorescent protein are fused to bait and prey proteins, respectively. Interaction between these proteins brings the split fragments together, restoring fluorescence emission upon excitation.

Fluorescence co-localization spectroscopy provides a valid approach to determine whether an uncharacterized protein condensate belongs to stress granules/P-bodies. A mRNP (messenger ribonucleoprotein) is a dynamic, multifunctional complex composed of mRNA and associated proteins (RNA-binding proteins, or RBPs). It governs mRNA metabolism from synthesis to degradation. Both stress granules and P-bodies are mRNP-related membraneless organelles [3]. When translation initiation is blocked, mRNPs cease to translate and assemble into P-bodies. mRNPs in stress granules are those preparing to reenter translation [46]. Checking whether these condensates are P-bodies or stress granules helps infer their possible functions, and fluorescence co-localization imaging with specific stress granule and P-body markers can be used to study these mRNP condensates. Jin et al. found membraneless granules concentrated by glycolytic proteins [47]. They termed the membraneless granules as a ‘glycolytic body’ or ‘G body’. Pfk2p is one of the components of a G body. They found that Pfk2p co-localized with Pab1, a component of stress granules. Under glucose deprivation, Pab1 and Pfk2p remained diffused. Under hypoxia, Pab1 diffused, and Pfk2p formed foci. They also co-localized Pfk2p with Edc3, a component of P-bodies. Edc3 formed foci under glucose deprivation or hypoxia. Pfk2p located adjacent to, but not overlapping with, Edc3 under hypoxia. The results showed that Pfk2p is not a component of either stress granules or P-bodies. Saad et al. tested the foci-forming condition of Cdc19, Pab1, and Edc3 [22]. They tested the condition upon addition of rapamycin, CHX, azide, hyperosmotic shock, H_2_O_2_ treatment, heat shock, and alpha-factor treatment. They also tested whether Cdc19 could co-localize with Pab1 or Edc3. The foci-forming condition of Cdc19 and Pab1 were similar, and they were found to be co-localized. The results showed that Cdc19 was one of the components of stress granules.

Some researchers have tested whether certain different proteins can condense into the same granule. O’Connell et al. mentioned that proteins that could co-localize might have similar functions [33]. Jin et al. identified 33 candidates that could form punctate foci under hypoxia [47]. They performed co-localization assays on these candidates with Pfk2p to find out whether they were components of G bodies. From their results, only two of those candidates could not co-localize with Pfk2p; therefore, 31 candidates were components of G bodies. Utsumi et al. used co-localization spectroscopy to identify the peptide fragments that lead to the formation of a ‘metabolic proteins transiently assembling (META) body’ [48]. They identified four fragments (SC1: 33–74 a.a.; SC2: 129–158 a.a.; SC3: 217–243 a.a.; SC4: 373–404 a.a.) inducing Cdc19 foci formation within META bodies. To test artifactual condensation, SC2/SC3 were fused to condensate inert Adh1, alongside FUSN/Sup35-Adh1 controls. Hypoxia triggered foci formation in SC2/3-Adh1 fusions that co-localized with Cdc19 and partially with Eno2p (another META component), whereas FUSN/Sup35-Adh1 foci showed no Cdc19 co-localization. These results confirm that SC2/3 specifically targets proteins to META bodies.

**Figure 1:**
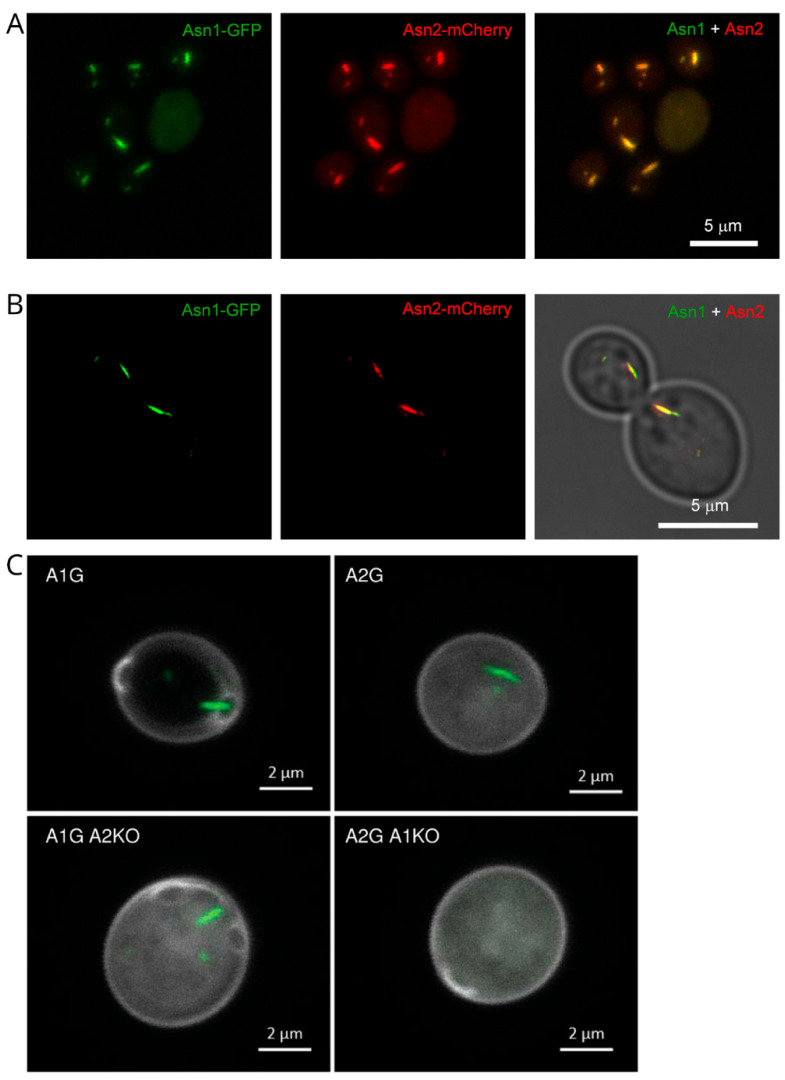
Fluorescence co-localization experiments of Asn1 and Asn2 (adapted from Zhang et al. 2018 with open copyright) [49]. (**A**,**B**) Asn1 and Asn2 could co-localize in yeast cells, shown in confocal microscopy (**A**) and STED (**B**). (**C**) Confocal images for yeast strains of Asn1-GFP, Asn2-GFP, Asn1-GFP with *ASN2* gene knocked out, and Asn2-GFP with *ASN1* gene knocked out. When the *ASN2* gene was knocked out, Asn1 could still form cytoophidium. When the *ASN1* gene was knocked out, Asn2 failed to form cytoophidium.

Fluorescence co-localization spectroscopy can also observe the co-assembly of filaments. Zhang et al. observed the co-assembly of Asn1p and Asn2p [49] (Figure 1). Asn1p co-localized with Asn2p both in the cytoplasm and in the nucleus. They also found that Asn1-*Asn2*(knocked out) could form filaments, but Asn2-*Asn1*(knocked out) could not, indicating that *Asn1* influences Asn2 filamentation. Noree et al. also reached the same conclusion regarding the co-assembly of Asn1p and Asn2p [50]. Chang et al. observed the co-assembly of Ctps and IMPDH [41]. They found that IMPDH and Ctps were not mixed within filaments. The mixed cytoophidium was formed by two or more IMPDH and Ctps cytoophidium, with a tiny gap between IMPDH and Ctps.

The co-localization method can combine with single-molecule photobleaching counting to achieve stoichiometry measurement. Single-molecule techniques utilize the unique characteristics of individual molecules to uncover information obscured by traditional ensemble measurements [51]. Leveraging the characteristic stepwise photobleaching of single-molecule fluorescent labels, these techniques can be integrated with fluorescence co-localization methods to determine the relative stoichiometry of target proteins during assembly. Single-molecule FRET (smFRET) is further applicable for probing intra-molecular conformational dynamics or distance changes in inter-molecular interactions. Chang et al. employed co-localization single-molecule spectroscopy to observe the co-assembly of Ctps and P5CS [52]. At a 1:1 concentration ratio of Ctps and P5CS, both components formed into co-localized cytoophidium. With 2.5 times more Ctps than P5CS, Ctps formed scattered oligomers, while a few P5CS co-polymerized with Ctps. Conversely, when the ratio of P5CS to Ctps was reversed, similar results were observed. The binding stoichiometry between Ctps and P5CS could be quantified using single-molecule photobleaching counting. Photobleaching steps ratio revealed stoichiometry. Within the buffer containing CTP, MgCl_2_, and glutamate, the estimated combining ratio of Ctps and P5CS was 2:1.

### 2.3. Methods Based on Fluorescence Photobleaching

**Figure 2:**
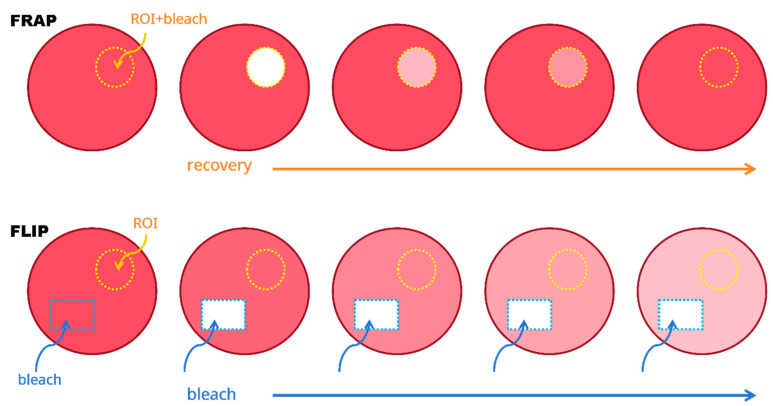
Schematic representations of FRAP and FLIP. For FRAP, the region of interest (ROI), shown in the yellow circle, is bleached once with an intense laser beam. The fluorescence recovery in the ROI is measured continuously. For FLIP, the region shown in the blue rectangle is the bleaching area. This area is under continuous bleaching. The fluorescence loss in the ROI (shown in the yellow circle) is measured continuously.

Beyond identification and monitoring interactions of protein condensates, fluorescence techniques have the advantage in time-resolved dynamical measurements in cellular environments. One of the most popular methods is utilizing the photobleaching property of conventional organic dyes. Photobleaching is employed to reveal the dynamics of the fluorophore within a target region. Two methods utilize photobleaching to monitor the dynamics of a given region: fluorescence recovery after photobleaching (FRAP), and fluorescence loss in photobleaching (FLIP) [53]. These two methods are complementary: in FRAP, the region of interest (ROI) is bleached; in FLIP, the region outside the ROI is bleached. The principles of FRAP and FLIP are shown in Figure 2.

In FRAP, the ROI is bleached once. The fluorescence molecules in this target region are exposed to a brief, intense pulse from a high-power laser, resulting in irreversible bleaching of the fluorophore in this region. Consequently, the fluorescence intensity of this target region decreases. After the bleaching event, the fluorescence molecules outside this region replenish the target area through diffusion. As a result, the fluorescence intensity of this region increases with a characteristic time scale determined by the viscosity of the subcellular environment [54]. Therefore, quantitative FRAP experiments provide the relative mobility of these molecules. Based on the mobility difference in these molecules, different phases or condensates can be distinguished [26,55]. We can also observe composition exchange with the surrounding environment using FRAP [22,26,56].

Saad et al. performed FRAP experiments to observe the formation and composition of Cdc19 condensate [22]. They detected no fluorescence recovery of Cdc19-GFP from aggregates with cytosolic Cdc19-GFP or within aggregates themselves. These findings indicate that the formation of Cdc19 condensate is caused by insoluble, solid-like aggregates.

Chang et al. employed FRAP to check whether proteins building cytoophidium have an active turnover [41]. They measured the fluorescence intensity of an individual cytoophidium at several time points. The signal recovery of Ctps-GFP was faster than IMPDH-GFP. The fluorescence intensity recovered evenly, and no detectable change in length of the unbleached parts at the two ends was found. The results suggested that the cytoophidium may continuously renew its subunits.

Fuller et al. used FRAP to explore whether G bodies could exchange with cytoplasm, and measured recovery kinetics of some of the G body components [56]. They photobleached the whole G body, and recorded the fraction of fluorescence recovery over time. They fitted the relationship between fluorescence recovery and time. Pfk2 had a very weak recovery, and the half time of recovery was on the order of minutes. Fba1 had more recovery than Pfk2, but the half time of recovery was similar to Pfk2. Eno2 also recovered, but it showed a different dynamic: the curve of fluorescence recovery to time seemed to be linear instead of exponential. They also tested whether RNA influenced G body dynamics using FRAP. After depletion of RNA from G bodies, more recovery of Pfk2 was observed, indicating that in the absence of RNase, Pfk2 associated with G bodies more stably.

In FLIP, a region outside the ROI undergoes repeated bleaching. Fluorescence loss of the ROI is monitored during the bleaching event [53]. If the fluorophores outside the region diffuse into the bleaching region, these fluorophores will eventually bleach, causing a decrease in fluorescence intensity outside the bleaching region. In contrast to FRAP, FLIP experiments focus on connectivity between different compartments, especially remote compartments [57].

Suresh et al. used FLIP to determine the dynamics in fatty acid synthetase (FAS) foci [58]. A small region of stationary-phase cell distant to FAS foci was continuously bleached, and they monitored the fluorescence intensity of the FAS foci. The fluorescence intensity of FAS foci dropped rapidly over the period of bleaching, showing that the exchange of FAS molecules between the foci and cytosol was very fast.

### 2.4. Super-Resolution Imaging

Conventional fluorescence microscopy has a lower resolution than electron microscopy due to the wavelength and diffraction limitation of light. Several approaches have been used to improve the resolution of fluorescence microscopy, such as multiphoton fluorescence [59], stimulated emission depletion (STED) [60], and saturated structured-illumination microscopy (SSIM) [61]. In 2006, a number of revolutionary approaches, including PALM [62] and STORM [63], were developed to improve the resolution of fluorescence microscopy. PALM/STORM leverages the photoswitching behavior of specific fluorescent probes [64] and employs centroid localization [65] to achieve super-resolution imaging beyond the diffraction limit. Besides fluorophore selection, PALM/STORM principally relies on post-imaging computations and imposes lower demands on existing hardware compared to some other super-resolution techniques. Consequently, they are among the most widely adopted super-resolution imaging methods. With super-resolution approaches, such as PALM/STORM, the resolution of fluorescence microscopy can be improved to 10–20 nm.

Super-resolution fluorescence microscopy enables high resolution imaging for in vivo experiments. For example, Prouteau et al. observed TORC1 structure in vivo at nanometer scale using STORM [30]. They categorized the clusters of reconstructed STORM images into three groups: no apparent organization, ring-like shape, and rod-like shape. The ring-like assembly had an average diameter of 115 nm, and the rod-like assembly reached an average diameter of 98 nm and length of 500 nm. The result suggested that TORC1 may form hollow tubule structures with a hole diameter around 100 nm in vivo.

Furthermore, super-resolution fluorescence microscopy renders studying co-assembly much easier, as different proteins can be readily labeled with distinct fluorescent protein tags. Chang et al. used STED to resolve the ultrastructure of Ctps and IMPDH mixed cytoophidium, and found that there was a gap between Ctps and IMPDH filaments [41]. Intriguingly, this implies that they did not interact directly with each other. Zhang et al. used STED and confirmed that Asn1 co-localized with Asn2 [49]. Li and Liu researched five cytoophidia-forming proteins (Asn1, Bna5, Ctps/Ura7, Glt1 and Prs5) using super-resolution live-cell imaging [66]. They found that Asn1/Ura7, Prs5/Ura7, Prs5/Ura7/Asn1, and Prs5/Ura7/Asn1/Glt1 could form cytoophidia complex, and Ura7 existed in every complex. Furthermore, the cytoophidia complex formed in the sequence of Prs5, Ura7 and Asn1, while Glt1 showed a very low abundance.

## 3. Electron Microscopy for Observing Protein Filaments

The advantages of fluorescence microscopy (FM) include live-cell compatibility, time-resolved dynamics capabilities, and a wide range of fluorescent proteins or other immuno-tagging probes for specific biomolecule labeling. However, fluorescence microscopy fails to distinguish protein filaments with different variants or isoforms due to limited spatial resolution. Electron microscopy (EM), on the other hand, reaches a much higher spatial resolution than FM. Although EM requires a series of sample treatments (e.g., fixation, freezing, and/or sectioning) that preclude it from live-cell and time-resolved imaging, and fails to resolve protein condensates, its ultra-high, sometimes atomic resolution makes it extremely powerful for protein filament studies. We can use electron microscopy to obtain detailed structural information of the filament, especially those proteins that have variants.

### 3.1. Negative-Stain Electron Microscopy

For protein samples, researchers use negative-stain electron microscopy [67]. The resolution of negative-stain electron microscopy reaches 1 nm. In positive-stain electron microscopy, the electron density of the stained sample structures is enhanced by the staining, making them appear black in the image, while the background remains bright due to the lack of stain. Negative staining involves the use of substances with high electron density in the stain solution to “embed” the sample with low electron density. As a result, the background appears dark in the image, while the sample seems transparent and bright. Negative staining is preferred for protein samples because it coats the specimen with heavy metals to indirectly highlight surface structures against a dark background without penetrating or distorting the delicate protein, whereas positive staining risks structural damage by directly binding heavy metals to the biomolecules, obscuring critical surface details and obstructing some inner details. For protein samples, uranyl acetate is commonly used as a staining material.

**Figure 3:**
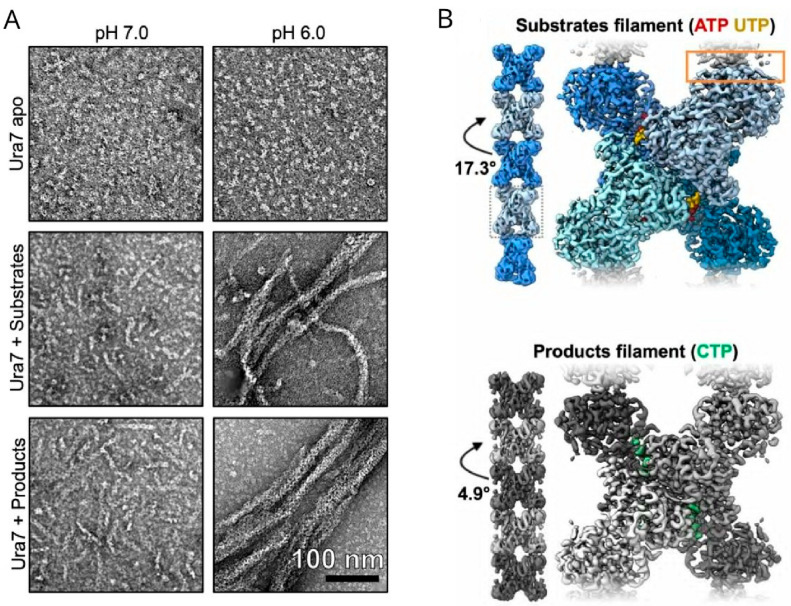
Negative-stain electron microscopy and cryo-EM images of Ura7 and Ura8 (adapted from Hansen et al. 2021 with open copyright) [72]. (**A**) The negative-stain electron microscopy images of Ura7. At pH 7.0, Ura7 apo, Ura7+Substrates, and Ura7+Products all showed short, single filaments. At pH 6.0, Ura7+Substrates and Ura7+Products formed larger polymers. (**B**) Cryo-EM images of Ura8+Substrates and Ura8+Products at pH 6.0. Ura8 reached a higher resolution than Ura7 at 2.8 Å (+Substrates) and 3.8 Å (+Products).

Using negative-stain electron microscopy, researchers can check the formation of these filaments in vitro and find out their detailed structure. Webb et al. observed the detailed filament structure of Pfk1 in vitro [68]. In control buffer containing 1mM ATP, Pfk1 was predominantly tetramers, and few were small aggregates. In the presence of 2mM F6P, Pfk1 assembled into filaments. Pfk1 filaments were stacked by tetramers that were related by a rotation of 221° and a translation of 83 Å. Zhang et al. checked whether P5CS could form filaments in vitro under different conditions [69]. Apo P5CS could hardly form filaments in vitro, but when substrates (ATP, NADPH, and glutamate) were added, P5CS could form filaments at 25 °C. Removing ATP or NADPH did not obstruct the P5CS filament formation, while removing glutamate abolished P5CS formation. They also found two basic subunits of P5CS filaments named cylinder A and cylinder B. The diameter of cylinder B was about 159 Å, and the periodicity of P5CS combining A and B was 165 Å. In comparison, the periodicity of substrate-bound P5CS was 106 Å. However, improved resolution was needed to reveal the difference between apo P5CS and substrate-bound P5CS. Anthony et al. used negative-stain electron microscopy to confirm whether some IMPDH2 mutants prevent IMPDH2 self-assembly [70]. They found that without ligands, mutant Y12A and R356A were in the form of octamers and tetramers, and could not form polymers. They also failed to observe Y12A and R356A polymers in the presence of ATP. In contrast, S275L could form polymers in both the absence and presence of ATP, suggesting that S275L polymerization propensity is an intrinsic consequence of the mutation. The relationship between IMPDH2 conformational change and catalysis activity were also explored using negative-stain electron microscopy. Four major classes of filament segments were identified: expanded, collapsed, bent, and ‘poorly aligned’. The bent conformation were well-resolved. The ‘poorly aligned’ segments were those without detailed features. They quantified the frequency of these classes in the absence or presence of ATP, GTP and substrates. The results showed that binding of either substrate shifts the conformational equilibrium toward the expanded state, and that GTP stabilizes the collapsed conformation. Calise et al. studied the two tissue-specific splice variants, IMPDH1(595) and IMPDH1(546), expressed in vertebrate retina [71]. Since residue S477 is preferentially phosphorylated in the dark, they investigated the influence of phosphorylation analog mutant S477D on filament assembly in vitro. They tested the condition of presence or absence of ATP or GTP. S477D completely prevented IMPDH1(595) assembly under all conditions. IMPDH1(546)-S477D could still form higher-order assembly under the presence of ATP or GTP. S477D also partially prevented self-assembly of the canonical IMPDH1(514). To gain more detailed information of how the S477D mutant affects the inhibited and active interfaces of IMPDH1(546), data can be analyzed from Cryo-EM map resolved ~3 Å. Applications of cryo-EM technique will be discussed in the following section. Using EM, Hensen et al. examined the structural conformations of *S. cerevisiae* Ura7 in three states: apo-form (substrate-free), substrate-bound, and product-bound [72] (Figure 3A). Notably, the apo-form failed to assemble filaments at any tested pH. In contrast, both substrate-bound and product-bound Ura7 formed short individual filaments at pH 7.4, while assembling into larger filamentous structures at pH 6.0. These observations reveal pH-dependent filament formation in yeast Ura7.

For proteins that can form different types of filaments, electron microscopy helps distinguish these filaments. For example, Hunkeler et al. found three types of acetyl-CoA (ACC) filaments [73]. The first type of filament was unbranched filaments induced by allosteric activator citrate. This type of filament is the most active form. Addition of excessive palmitoyl-CoA to preformed ACC-citrate filaments could inhibit ACC, and induced a transition to the second type of filament, ACC-citrate^palm^. BRCT binding to phosphorylated ACC yields the third type of filament: ACC-BRCT. The third type of filament has a different structure from ACC-citrate and ACC-citrate^palm^. This type is the most inactive form. Regulation activity is controlled by change in different types of filaments.

Electron microscopy can observe the detailed structure of amyloid. Cereghetti et al. observed Cdc19 amyloid using electron microscopy [74]. Cdc19 amyloid core peptide formed fibrillar aggregates at pH 5.8. This pH value corresponds to starved or heat-shocked yeast cells. Cdc19 core peptide remained soluble at pH 7.4, corresponding to the pH of growing yeast cells. The results showed pH-dependent Cdc19 amyloid core aggregation.

### 3.2. Cryo-EM

There are still some disadvantages of negative-stain electron microscopy. A significant disadvantage is the structural compromise of protein samples: heavy metal coating and air-drying potentially introduce artifacts obscuring the native architecture. To overcome these disadvantages and better preserve the native structure of protein filaments, cryo-EM was developed. Jacques Dubochet aimed at developing methods for making frozen specimens. He found that amorphous ice could be formed using pure water through a sufficiently fast freezing process in 1981 [75] and applied this method to many biological samples [76]. The amorphous ice could preserve the native states of specimen in the solution, and reduce radiation damage from high-energy electrons. Henderson et al. determined the transmembrane helices at 7 Å using glucose solution to its native state in 1975, which was the technical limit they could achieve at room temperature at that time [77]. In 1990, Henderson et al. determined the structure of bacteriorhodopsin at 3.5 Å using the sample preparation method developed by Dubochet [78]. Joachim Frank’s work aimed at developing an algorithm to process EM images [79,80]. Using his algorithm, 3D structures of these proteins are reconstructed. To further improve the capability, cryo-electron tomography (cryo-ET) was developed, which combined cryo-EM and tomography [81]. Cryo-ET is mainly used to study cellular ultrastructure.

In cryo-EM, the sample is rapidly frozen, becoming embedded in a thin layer of vitreous ice. This process, known as vitrification, immobilizes the sample in a near-physiological, hydrated state. Furthermore, cryo-EM reaches a resolution of Angstrom, superior to the ~1 nm resolution of negative-stain EM. While negative-stain EM cannot distinguish double-stranded filaments from single-stranded filaments, cryo-EM helps researchers distinguish whether these filaments are double-stranded. Furthermore, detailed structures help researchers analyze the interactions between key residues for filament formation.

Most of the works we reported in the previous section have also carried out cryo-EM imaging besides negative-stain EM. For example, Hunkeler et al. explored detailed ACC filament structure variants using cryo-EM [73]. For ACC-citrate filament, two BC domains form a dimer, which is considered necessary for BC activity. The CT domain serves as a docking platform for the BCCP domain in the enzyme’s resting state, facilitating spatial organization critical for catalytic efficiency. The BCCP domain can rotate between the BC domain and CT domain. For ACC-citrate^palm^ filament, the BC dimer destabilized, causing a reduction in ACC activity. For ACC-BRCT filament, the BC domain is monomeric, leading to the inactive form of ACC. The BCCP domain is unable to reach any of the active site. The structure difference in different ACC filaments leads to different activity.

Hansen et al. used cryo-EM to further explore the structure basis for pH-sensitive Ctps filament formation [72] (Figure 3B). Since the resolution of Ura8 could reach 2.8 Å (substrate) and 3.8 Å (product) using cryo-EM, they chose to analyze Ura8. From the cryo-EM structure, Ura8 assembled as stacked tetramers. The residue H360 interacted with D370, which can be stabilized by protonation of H360 at low pH. This indicated that residue H360 is responsible for the pH-sensitivity of Ctps filaments. The principal distinction between substrate-bound and product-bound Ura8 lies in their conformational states. Specifically, a ~7° rotation of the glutaminase domain relative to the amido-ligase domain distinguishes the active versus inhibited conformations. Cryo-EM structures demonstrate that product-bound filaments adopt the canonical inhibited conformation, whereas substrate-bound filaments exhibit an intermediate conformation between active and inhibited states. Collectively, these structural data indicate that filament formation stabilizes Ura8 in an inactive enzymatic state.

Stoddard et al. resolved the detailed structure of Glk1 using cryo-EM [31]. Before using cryo-EM, they used negative-stain EM and found out its helical filament structure. The 3.8 Å resolution from cryo-EM revealed the two-stranded, antiparallel filament structure of Glk1.

Anthony et al. used cryo-EM to determine whether human IMPDH2 could adopt a collapsed conformation similar to *Ashbya gossypii* IMPDH [70]. They reached a resolution of 8.7 Å. Comparing the human GTP-bound Y12A IMPDH2 with the *Ashbya gossypii* GDP-bound IMPDH2 crystal structure, they found that in the presence of GTP, human IMPDH2 is in the collapsed conformation.

Calise et al. used cryo-EM to gain detailed structures of IMPDH isoforms [71]. They resolved a 3.1 Å structure of IMPDH1(595)-S477D in the presence of GTP, ATP, IMP and NAD+. They aligned S477D and wild type tetramers on chain A and observed a 3° shift in chain C, indicating that IMPDH1(595)-S477D free octamer adopts a bowed conformation. Then they resolved a 3.3 Å structure of IMPDH1(546)-S477D compressed octamer within a filament in the presence of GTP, ATP, IMP, and NAD^+^. Aligning with the S477D and wild type, little difference was found. A 2.4 Å structure of IMPDH1(546)-S477D and IMPDH1(546)-WT was resolved in the active state (in the presence of ATP, IMP and NAD^+^). Under this resolution, they observed a 3° shift in the protomer on the opposite side of the octamer. Another 2.1 Å interface-centered structure of IMPDH(546)-S477D was compared with its IMPDH(546)-WT counterpart. IMPDH(546)-S477D was found in an intermediate conformation between bowed and flat. These results suggested that in the presence of GTP, the enzyme preferred the compressed, bent state.

## 4. Correlative Light/Electron Microscopy for Observing Protein Filaments

Correlative light and electron microscopy (CLEM) is a powerful imaging technique that integrates information obtained on fluorescence microscopy and electron microscopy [82]. Ever since the development of EM, there had been efforts to combine FM with EM, although such attempts did not generate sufficient impact. Several technical developmental milestones, including the discovery of genetically encoded fluorescent proteins [83,84], the invention of super resolution FM [62,63], and the advancement of single-particle cryo-EM [85], were instrumental. CLEM can directly and quickly localize the target filament. There are different workflows for executing CLEM; for example, to achieve both live-cell imaging or a more physiological environment with EM, one can complete FM before freezing treatments of the specimen. Recently, super-resolution fluorescent proteins that can sustain freezing have been developed [86], so in situ cryo-EM/ET and SR FM has become feasible.

**Figure 4:**
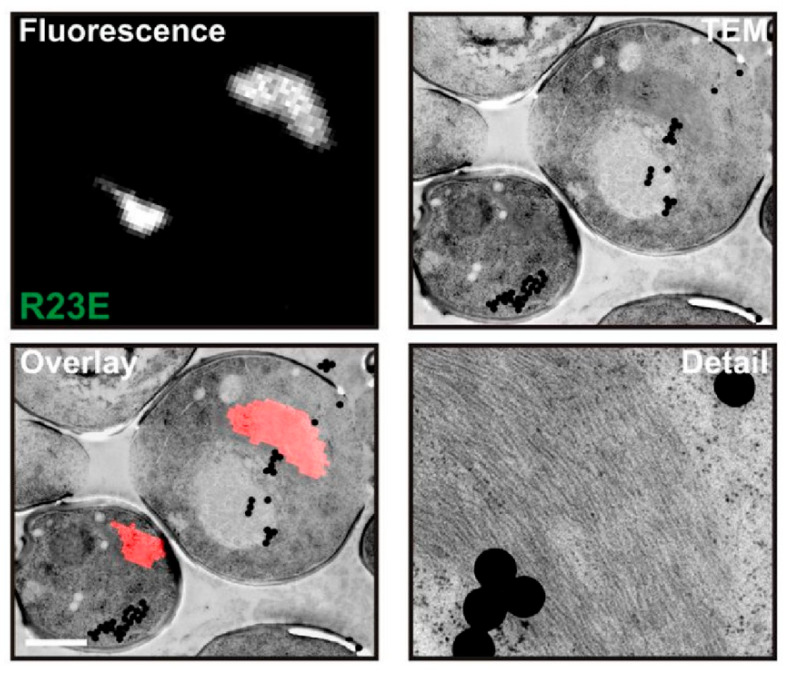
Correlative light electron microscopy (CLEM) used in mCherry-tagged R23E mutant of Gln1 (adapted from Petrovska et al. 2014 with open copyright) [28]. Fluorescence images aligned with TEM images. A large number of filaments could be seen in the TEM images. These filaments aligned into higher order bundles.

Petrovska et al. employed CLEM to analyze cells overexpressing Gln1 mutants after identification of these filaments using fluorescence microscopy [28] (Figure 4). Using EM helps reveal the ultrastructure of Gln1, such as their assembly mechanism, and CLEM allows regions found in FM to relocate in EM. The FM images of Gln1 mutants were precisely overlaid with corresponding EM images. The EM images revealed numerous filaments that were laterally aligned into higher order bundles. The side-by-side bundling pattern aligned with the filament growth pattern, which was primarily longitudinal but also involved a degree of circumferential expansion over time. Their results showed that Gln1 assembled into filaments by a back-to-back stacking mechanism.

Thomas et al. used CLEM to further obtain the ultrastructure of IMPDH2 filaments after observing these filaments using immuno-electron microscopy and immunofluorescence [87]. They ensured that the GFP-tagged filaments found in fluorescence microscopy were the same as those found in immuno-electron microscopy. They found the macroscopic ultrastructure of IMPDH macrostructures.

Paukštytė et al. used CLEM to better understand the nature of Glt1 assembly [88]. After localizing the Glt1 filament using fluorescence microscopy, they found the corresponding electron graphs. These electron graphs showed that Glt1 polymers were bundled filaments, indicating that Glt1 assembly might occur at the polymer ends.

Suresh et al. used correlative fluorescence microscopy and electron tomography (using electron tomography instead of electron microscopy) [58]. They correlated 10 Fas1-mCherry foci with corresponding electron tomograms, and verified that Fas1 foci represent ribosome-free zones in the cytosol.

Processing CLEM data is extremely demanding for computer hardware, since often hundreds of gigabytes to several terabytes of CLEM data are collected [89,90]. Therefore, it is hard for many studies to employ CLEM to obtain further information about these protein filaments. Using fluorescence microscopy and electron microscopy separately cannot ensure fluorescent targets; EM structures are homologous, but are available for more researchers.

## 5. Concluding Remarks

In this review, we introduced a methodological framework to study protein condensates or filaments, from foundational imaging techniques to cutting-edge single-molecule and correlative imaging technologies. For fluorescence methods, we introduce localization screening, fluorescence co-localization spectroscopy, methods based on photobleaching, and super-resolution imaging. For electron microscopy, we introduce negative-stain electron microscopy, cryo-EM and the combined CLEM used in the field of protein condensates. We have documented how classical fluorescence microscopy (FM) and electron microscopy (EM) evolved into sophisticated tools like super-resolution FM (PALM/STORM), cryo-EM, and correlative light/electron microscopy (CLEM), collectively dismantling resolution barriers and enabling unprecedented spatial-temporal interrogation of condensate dynamics, stoichiometry, and ultrastructure. Depending on these methods, we can identify protein condensates or filaments, discover their potential functions, and investigate their dynamics. We summarize these methods in Table 1.

The case studies herein—spanning condensate or filament identification (e.g., Gln1 filaments), phase characterization (Cdc19 aggregates), structural elucidation (TORC1 toroids), and interaction mapping (CTPS-P5CS co-assembly)—underscore a critical paradigm: methodological selection must align with biological questions. For rapid in vivo dynamics, FM-based approaches remain indispensable; for atomic-resolution architecture, cryo-EM is unparalleled, while CLEM bridges these scales for contextualized analysis. This toolbox empowers researchers to navigate methodological trade-offs between resolution, throughput, and physiological relevance.

Knowledge of protein condensates or filaments demands further scrutiny. Most protein condensates or filaments are found in budding yeast, and we do not know as much about their counterparts in other species. Furthermore, some protein condensates or filaments are found by screening, but we know little about their physiological function without further research. Especially, what are the determining factors of condensate or filament formation? Is it specific for metabolic-related enzymes? Protein condensates or filaments formed under distinct conditions exhibit diverse functions. Certain undiscovered functions of these condensates or filaments may hold potential applications. Thus, we anticipate that the reviewed methods and their applications will facilitate the elucidation of unexplored condensates or filaments and their yet-to-be-characterized biological roles.

## Figures and Tables

**Table 1:** The techniques introduced in this review.

Techniques	Function	Application (Condensate or Filament)
Localization screening	Find out condensate or filament-forming proteins [6,7,34,35]	Condensate and filament
Fluorescence co-localization spectroscopy [42]	Determine whether protein condensate belongs to p-body or stress granule [22,47]	Condensate
	Determine whether different proteins condense into the same granule [47,48]	
	Observe co-assembly of different filaments [41,49,50]	Filament
FRAP [53]	Measure the mobility of condensates; distinguish different phases or condensates [41,56]	Condensate
	Observe composition change with surrounding environment [22]	
FLIP [53]	Reveal connectivity between different compartments [58]	Condensate
STORM [62,63]	Higher resolution for in vivo imaging [30,41,49,66]	Condensate and filament
Electron microscopy	Higher resolution for in vitro experiments [68,69,70,71,72,73,74]	Filament
CLEM	Combine fluorescence microscopy data with electron microscopy data [28,58,87,88]	Filament

## Data Availability

The data presented in the study are openly available in Figure 1 at Ref. [49]; in Figure 3 at Ref. [72]; in Figure 4 at Ref. [28].

## Abbreviations

The following abbreviations are used in this manuscript:

CLEM	Correlative light and electron microscopy
EM	Electron microscopy
FLIP	Fluorescence loss in photobleaching
FRAP	Fluorescence recovery after photobleaching
FM	Fluorescence microscopy
META	Metabolic enzymes transiently assembling
PALM	Photoactivated localization microscopy
POI	Protein of interest
ROI	Region of interest
STED	Stimulated emission depletion
STORM	Stochastic optical reconstruction microscopy
TORC1	Target of rapamycin complex 1.
